# Fischer 344 and Lewis Rat Strains as a Model of Genetic Vulnerability to Drug Addiction

**DOI:** 10.3389/fnins.2016.00013

**Published:** 2016-02-09

**Authors:** Cristina Cadoni

**Affiliations:** ^1^Institute of Neuroscience, Cagliari Section, Department of Biomedical Sciences, National Research Council of ItalyCagliari, Italy; ^2^Centre of Excellence “Neurobiology of Dependence”, University of CagliariCagliari, Italy

**Keywords:** Lewis rats, Fischer 344 rats, reward, addiction, impulsivity, HPA axis

## Abstract

Today it is well acknowledged that both nature and nurture play important roles in the genesis of psychopathologies, including drug addiction. Increasing evidence suggests that genetic factors contribute for at least 40–60% of the variation in liability to drug dependence. Human genetic studies suggest that multiple genes of small effect, rather than single genes, contribute to the genesis of behavioral psychopathologies. Therefore, the use of inbred rat strains might provide a valuable tool to identify differences, linked to genotype, important in liability to addiction and related disorders. In this regard, Lewis and Fischer 344 inbred rats have been proposed as a model of genetic vulnerability to drug addiction, given their innate differences in sensitivity to the reinforcing and rewarding effects of drugs of abuse, as well their different responsiveness to stressful stimuli. This review will provide evidence in support of this model for the study of the genetic influence on addiction vulnerability, with particular emphasis on differences in mesolimbic dopamine (DA) transmission, rewarding and emotional function. It will be highlighted that Lewis and Fischer 344 rats differ not only in several indices of DA transmission and adaptive changes following repeated drug exposure, but also in hypothalamic-pituitary-adrenal (HPA) axis responsiveness, influencing not only the ability of the individual to cope with stressful events, but also interfering with rewarding and motivational processes, given the influence of corticosteroids on dopamine neuron functionality. Further differences between the two strains, as impulsivity or anxiousness, might contribute to their different proneness to addiction, and likely these features might be linked to their different DA neurotransmission plasticity. Although differences in other neurotransmitter systems might deserve further investigation, results from the reviewed studies might open new vistas in understanding aberrant deviations in reward and motivational functions.

## Introduction

Reward is an essential function for the survival of individuals and for species perpetuation. In addition, it is a fundamental concept for discussions on drug abuse and addiction. Thus, in most theories of addiction altered sensitivity to either drug-reward, or to reward in general, contributes to or results from drug taking behavior (Di Chiara, 1998; Everitt et al., 2001; Robinson and Berridge, 2001, 2008; Di Chiara et al., 2004; Koob, 2006, 2013; Everitt and Robbins, 2013; Wise and Koob, 2014). This comes from the fact that drugs of abuse interact directly with the reward system, whose normal purpose is to provide the organism with the ability to recognize biologically relevant events occurring in the environment.

It has been clearly shown that both nature and nurture model the individual phenotype contributing both to successful adaptation to different environmental events but also playing important roles in the genesis of psychopathologies, included drug addiction, as demonstrated by family, twin, and adoption studies (Crabbe, 2002; Tsuang et al., 2004). While the role of genetic background in the onset of psychopathologies is well recognized, it remains difficult to find genes for these disorders. The main reason for this is the complex interaction of multiple genes of small effects with a variety of environmental factors. Thus, gene-environment interaction might provide one explanation for inconsistent findings in genetic association studies between genetic markers and mental disorders, as well as variability in heritability estimates for the same disorders (Kendler and Eaves, 1986; Ottman, 1996; Yang and Khoury, 1997; Rutter and Silberg, 2002; Dick et al., 2015).

Among the different approaches available to investigate genetic contribution to behavioral disorders, the use of genetic animal models has great potential as demonstrated by the advancement of our knowledge especially in the field of substance addiction (Crabbe, 2002). Compared to human genetic studies animal models offer the advantage of studying a particular behavioral phenotype in inbred strains under controlled experimental conditions through manipulations that, for ethical reasons, are not possible in humans. Needless to say animal models cannot reproduce the complex human condition (Stephens et al., 2010), but nonetheless might provide new insights guiding future human genetic studies.

When reasoning about reward mechanisms, and thus about addiction, the focus of discussions has been the dopamine (DA) system and in particular the mesolimbic DA system. Once it was true that all roads led to Rome, recently it has been said that all roads lead to dopamine, since most of the scientific literature recognizes that the major reward neurotransmitter pathway in the brain is indeed the dopamine system (Blum et al., 2012). For this reason the majority of studies aimed at understanding the molecular processes leading to dependence have been mainly focused on this system. This has been our aim too, trying to link genetic vulnerability to drug addiction to innate differences in DA system functionality and plasticity.

The present review will present evidence pointing to the validity, or suitability, of the Fischer 344 (F344) and Lewis (LEW) model as an investigative tool for understanding how genetic vulnerability is translated in neurochemical terms, thus providing new targets for genetic human studies. First, results from behavioral studies will be presented, highlighting differences in sensitivity to the rewarding and reinforcing properties of different drugs of abuse. Then, evidence concerning DA transmission functionality, by using *in vitro* and *in vivo* techniques, will provide a likely explanation for the previously reported behavioral differences between strains. Since one of the major differences between these strains is their different sensitivity to stress, differences in their hypothalamic-pituitary-adrenal (HPA) axis activity will be shown that might help to explain their different vulnerability to addiction, given the known ability of this system to interfere with rewarding and motivational processes. Moreover, differences between strains in other neurotransmitter systems, structural plasticity, and gene expression will be presented to further help in understanding the neurobiological basis of genetic vulnerability to drug addiction. When available, findings from genetic human studies, pointing to genes for proteins found to be differentially expressed in these strains, will be discussed to further strengthen the validity of this model, and increase our knowledge on genetic contribution to addiction.

## Behavioral studies

Early evidence of differential sensitivity of F344 and LEW strains to drugs of abuse was reported in 1988 by Suzuki et al. showing that ethanol serves as a strong positive reinforcer for LEW but as a weak reinforcer for F344 rats (Suzuki et al., 1988a) and that preference for morphine and codeine admixed food was higher in LEW as compared with F344 strain (Suzuki et al., 1988b). Behavioral studies on the effects of chronic morphine administration showed that LEW rats display a greater amount, but also a greater rate of reduction, of stupor across the 7 days of morphine treatment and show larger signs of opiate withdrawal following naloxone injection at the end of morphine treatment as compared with F344 strain (Mayo-Michelson and Young, 1992). However, F344 rats are highly susceptible to dependence on benzodiazepines (Suzuki et al., 1992), although severity of withdrawal signs following chronic pentobarbital is greater in LEW than in F344 rats (Suzuki et al., 1987). In this section several findings will be presented pointing to the LEW strain as more sensitive, compared with F344, to the rewarding and reinforcing properties of drugs of abuse.

### Conditioned place preference studies

Place conditioning is a procedure commonly used to measure the motivational effects of rewarding and aversive stimuli in laboratory rodents (Tzschentke, 1998, 2007; Bardo and Bevins, 2000; Huston et al., 2013). This paradigm is based on principles of Pavlovian conditioning, where an unconditioned stimulus (US, for example a drug) is repeatedly paired with a neutral environment characterized by distinctive cues (visual, tactile, and eventually, olfactory). By repeated pairing, the paired environment acquires motivational properties and becomes a conditioned stimulus (CS), approached or avoided depending on the nature of the US. Using this paradigm it has been demonstrated that most drugs of abuse induce conditioned place preference (CPP) reflecting their rewarding properties (see Tzschentke, 2007 for an extensive review).

Few CPP studies have been performed on F344 and LEW rats but most of them have demonstrated that morphine, heroin, cocaine, and nicotine are more rewarding in LEW than in F344 strain (Guitart et al., 1992; Kosten et al., 1994; Horan et al., 1997; Philibin et al., 2005; Grakalic et al., 2006; Cadoni et al., 2015), with the only exception being that of amphetamine CPP where F344 rats display preference for the drug paired compartment while LEW do not (Stöhr et al., 1998). While contrasting results with the above findings on morphine rewarding effects (Davis et al., 2007) might be explained by the nature, biased vs. unbiased, of the CPP procedure utilized, it should be underlined that different results might be obtained depending on the drug dose utilized (Kosten et al., 1994). This fact seems to be consistent with different effects of drugs of abuse on mesolimbic DA transmission depending on drug dose (Cadoni and Di Chiara, 2007). It seems that drugs stimulating DA transmission in the nucleus accumbens (NAc) shell to a greater extent, or showing a preferential stimulation of DA transmission in the shell compared with the core of NAc, induce a higher preference for the compartment previously paired with the drug (see Cadoni and Di Chiara, 2007 for a discussion). This is consistent with the role of DA in the NAc shell in incentive learning (Fenu et al., 2006; Spina et al., 2006).

However, when using the CPP paradigm for testing drug reward it should be kept in mind that results from these studies might be affected by adaptive changes in mesolimbic DA transmission, occurring after repeated drug exposure during conditioning (e.g., biochemical sensitization), that might be different in the two strains (Cadoni et al., 2015). While sensitization of DA and GLU transmission in the NAc core might delay extinction of CPP, given the role played by DA and GLU transmission in this area in drug reward memory (Wang et al., 2008; Li et al., 2011; Kobrin et al., 2015), sensitization of DA transmission in the NAc shell might increase the incentive value of the drug paired compartment (Bossert et al., 2007, 2012; Ito and Hayen, 2011) leading to increased CPP.

Moreover, the different hypothalamic-pituitary-adrenocortical (HPA) axis responsiveness of the two strains might play also a role in the effects of psychostimulants in CPP paradigm. Thus, it is known that the HPA axis has a role in the behavioral and biochemical effects of psychostimulants (Rivet et al., 1989; Cole et al., 1990) and that stress cross-sensitizes with psychostimulants (Deroche et al., 1995; Kalivas and Stewart, 1995; Prasad et al., 1995; Rougé-Pont et al., 1995; Cadoni et al., 2003). This cross-sensitization has been reported to be associated with an increased DA transmission in the NAc core (Cadoni et al., 2003), as observed in the case of drug-induced sensitization (Cadoni and Di Chiara, 1999; Cadoni et al., 2000; Vanderschuren and Kalivas, 2000). Thus, differences between strains in HPA axis reactivity to psychostimulants might play a role in differences found in psychostimulant CPP studies. It might be hypothesized that greater amphetamine CPP observed in F344 vs. LEW strain is related to their higher HPA axis reactivity to the same drug, through an influence on DA transmission in the NAc core.

### Operant self-administration studies

In addition to non-operant self-administration studies reported above, operant self-administration (SA) studies have provided further evidence of the vulnerability of LEW rats to the reinforcing properties of several drugs of abuse.

A wide range of drug self-administration techniques has been developed to model specific aspects of addiction (Panlilio and Goldberg, 2007). Thus, while fixed ratio (FR) schedule of responding is a measure of the reinforcing properties of the drug, change in the effort required to obtain the reinforcer gives us a measure of the animal's motivation to work for obtaining that reinforcer, thus giving us more insight into the process of transition to addiction. In this regard, the progressive ratio (PR) schedule, utilized in several SA studies, is intended to better characterize the individual vulnerability of the animal based on the assumption that the greater is the animal's need for the drug the greater will be the effort the animal will put in to obtain the drug.

Another aspect of the procedure that has been used to mimic better the human condition of transition from casual use to addiction is the length of the session, as extended access (6 h or more) to the drug could model the ideal condition to unveil an escalation of drug intake suggestive of a development of addiction. By the use of manipulations of the SA conditions it is also possible to study the vulnerability of the individual to relapse, thus mimicking one of the more challenging aspects of drug addiction, through testing the ability of stress, drug-conditioned cues or the drug itself to reinstate drug seeking and taking behavior. To date, several SA studies have been performed on LEW and F344 rats and most of them point to the LEW strain as the vulnerable one or the “addiction prone” one.

Previous studies showed that LEW rats are more sensitive to the reinforcing properties of *ethanol*. It has been demonstrated that not only under FR1 but also under different FR schedules of responding (1, 2, 4, 8, and 16), and using different ethanol concentrations, LEW strain shows higher rates of responding, greater ethanol intake, and higher blood ethanol levels compared with F344 (George, 1987; Suzuki et al., 1988a).

With regards to *opiates* SA studies, LEW rats have been reported to maintain higher rates of lever pressing and to consume larger amounts of etonitazene, as compared with F344 rats, in a operant self-administration paradigm where the schedule of responding was increased from FR-1 up to FR-8, thus demonstrating differences in opioid reinforcement between strains (Suzuki et al., 1992). LEW rats acquire SA behavior faster, show higher rate of responding for morphine and heroin and show faster adaptation to the switching of the ratio schedule of responding for heroin (Ambrosio et al., 1995; Martín et al., 1999, 2003; Sánchez-Cardoso et al., 2007; Di Chiara et al., 2013), irrespective of the dose used (García-Lecumberri et al., 2011), and reach higher breaking points in PR schedule conditions (Martín et al., 1999, 2003; García-Lecumberri et al., 2011; Di Chiara et al., 2013). LEW rats show also shorter inter-response lever presses in variable interval schedule of responding (Martín et al., 2003), thus suggesting higher motivation and more compulsive behavior of the LEW strain in drug taking.

A striking demonstration of the greater vulnerability of LEW strain to addiction has been provided by the study of Picetti et al. (2012) who, using an extended access (18 h/day) paradigm that allowed the animal to choose between doses, showed that in these conditions LEW rats escalate their heroin intake overtime while F344 rats do not, thus mimicking the human condition of heroin addiction. Moreover, they demonstrated LEW preference for the higher and F344 preference for the lower doses of heroin, most likely due to the differences in basal opioidergic tone in the two strains and/or to differences in density and functionality of μ opioid receptors (MORs) in the two strains after opiate self-administration (Sánchez-Cardoso et al., 2007; Picetti et al., 2012).

Other studies investigating differences between strains in *cocaine* SA, have reported some conflicting results. It has been reported that LEW rats acquire cocaine SA faster and at lower doses under a FR1 during 2 or 6 h/daily sessions, with no differences in BP between strains in PR schedules (Kosten et al., 1997; Freeman et al., 2009). Conversely, F344 rats have been reported to self-administer more cocaine than LEW or SD rats under both fixed and progressive ratio schedule of responding (Kosten et al., 2007; Freeman et al., 2009) with more ineffective lever presses observed in F344 rats, a possible measure of craving. A more recent study by Picetti et al. (2010) provided a possible explanation for these discrepancies by using an extended access paradigm (18 h/day) allowing animals a dose choice. The results obtained showed the clear preference of LEW rats for the two higher doses and a high percentage of animals (35%) escalating to the highest unit dose and increasing their total amount of cocaine intake over days. Notably, while LEW rats started by self-administering less cocaine than F344 rats, they escalated their cocaine intake overtime, while F344 rats maintained a constant intake across the exposure period, thereby agreeing with a study that used a very different protocol (Freeman et al., 2009).

There is limited evidence regarding SA of other psychostimulants. Kruzich and Xi (2006b) showed that, in a limited access (2 h) condition and under FR1 schedule of responding, LEW rats display greater responding and higher *methamphetamine* intake, and emit more ineffective responses than F344 rats.

In another study, comparing 12 different inbred rat strains, LEW rats acquired SA faster and showed higher rate of responding during *amphetamine* SA than F344 rats, using different FR schedules of responding (FR1 to FR5), even when the dose was increased, but no difference was observed during extinction or reinstatement by amphetamine priming (Meyer et al., 2010).

An interesting study by Meyer and Bardo (2015) provided evidence of the effects of rearing conditions on *amphetamine* SA. When F344 and LEW rats are reared in social conditions (i.e., housed in groups without objects) LEW rats show higher amphetamine SA rate of responding, while these differences were not detected in enriched (i.e., housed in groups with objects) and isolated conditions, with enrichment buffering against SA, and isolation increasing SA in both strains. These results, while likely due to the different HPA activity of these strains, posit a fundamental concern in experimental research due to differences in breeding sources and different housing conditions used among research groups.

LEW rats are highly sensitive to *nicotine* reinforcement compared with F344, as previously suggested by CPP studies. While, Shoaib et al. (1997) found that neither LEW nor F344 rats acquire nicotine SA, following studies (Brower et al., 2002; Sharp et al., 2011), using an unlimited (23 h) as well as a limited (2 h) access paradigm to nicotine and low doses of nicotine (0.03 mg/kg), showed that LEW, but not F344 rats, acquire and maintain nicotine SA at different FR (1–4) schedules of responding and increase responses to increased effort requirements or decreasing drug reinforcement (dose). Moreover, a comparison of the reinforcing effects of nicotine in 6 inbred rat strains showed that LEW rats were the most sensitive while F344 rats were the least sensitive among the strains utilized (Chen et al., 2012). Also noteworthy is the finding from the same study about heritability of nicotine intake (0.64), which was very similar to that obtained in human studies (0.33–0.71) (Kendler et al., 1999; Maes et al., 2004; Vink et al., 2005; Ray et al., 2007).

Characterization of a phenotype vulnerable to addiction implies testing of reinstatement of drug seeking and taking behavior by different stimuli. This is usually performed in the SA paradigm after extinction training or sometimes after a period of forced abstinence (Fuchs et al., 2006; Reichel and Bevins, 2009; Millan et al., 2011).

Extinction is the reduction in drug seeking when the operant behavior is not reinforced by the delivery of the drug anymore. Few studies have evaluated extinction after psychostimulant and opiate SA in F344 and LEW strains. After *morphine* SA either a greater increase in responding in F344 than LEW rats or no differences between strains were observed (Ambrosio et al., 1995; Sánchez-Cardoso et al., 2007; Di Chiara et al., 2013). Extinction of *cocaine* SA showed no differences between strains (Miguéns et al., 2013) or greater responding during the 1st session in F344 rats and higher responding during the 2nd and the last session of extinction in LEW rats (Kruzich and Xi, 2006a). Following *methamphetamine* SA Kruzich and Xi (2006b) found greater responding during the 1st session of extinction in F344 compared with LEW rats, but F344 rats also showed increased responding on the inactive lever. However, it should be considered that extinction is an active learning process that might be affected by several factors other than the animal's motivation for drug seeking and more importantly does not actually mimic the human condition of abstinence thus lacking face validity (Sanchis-Segura and Spanagel, 2006). Moreover, there is evidence that extinction can ameliorate or reverse the neuroadaptations produced by chronic drug SA and/or by spontaneous abstinence (Millan et al., 2011).

In spite of several studies on morphine SA in F344 and LEW rats, only one study has investigated differences in reinstatement of opiate SA after extinction training (Di Chiara et al., 2013). This showed that LEW rats are more vulnerable than F344 rats to heroin priming reinstatement but not to drug conditioned cue-induced reinstatement, thus in agreement with a previous CPP study (Cadoni et al., 2015). In psychostimulant SA studies LEW rats showed higher rate of responding following cocaine or methamphetamine priming injections compared with F344 rats (Kruzich and Xi, 2006a,b; Miguéns et al., 2013). Table 1 summarizes the above results.

**Table 1:** **Results of drug self-administration studies in LEW and F344 rats**.

**Drug and schedule**	**Acquisition**	**Maintenance**	**Breaking point**	**Dose escalation**	**Extinction**	**Reinstatement**	**References**
**ETHANOL**							
FR1, 3, 4, 8, 16, 1 h	LEW > F344	LEW > F344	–	–	–	–	George, 1987; Suzuki et al., 1988a
**ETONITAZENE**							
FR1, 2, 4, 8, 1 h	LEW > F344	LEW > F344	–	–	–	–	Suzuki et al., 1992
**MORPHINE**							
FR1, 23 h;	LEW > F344	LEW > F344		–		LEW > F344	Ambrosio et al., 1995; Martín et al., 1999, 2003; Sánchez-Cardoso et al., 2007; García-Lecumberri et al., 2011
FR1, PR, 12 h;	LEW > F344	LEW > F344	LEW > F344		LEW = F344		Martín et al., 1999, 2003; García-Lecumberri et al., 2011
					F344 > LEW		Ambrosio et al., 1995
**HEROIN**							
FR1, 3, 5, 1 h;	LEW > F344	LEW > F344	LEW > F344		LEW > F344 (with cues)	LEW > F344	Di Chiara et al., 2013
PR, 4 h;					LEW = F344 (without cues)		
FR1, 18 h	LEW > F344	LEW > F344		LEW Yes F344 No			Picetti et al., 2012
**NICOTINE**							
FR1, 2 h	Neither acquired	–	–	–	–	–	Shoaib et al., 1997
FR1, 23 h	LEW > F344	LEW > F344					Brower et al., 2002; Sharp et al., 2011; Chen et al., 2012
**COCAINE**							
FR1, 2 h	LEW > F344	LEW > F344	–	–	–	–	Kosten et al., 1997
		F344 > LEW	F344 > LEW	–	–	–	Kosten et al., 2007
	LEW = F344	LEW = F344			LEW = F344	LEW > F344	Miguéns et al., 2013
	LEW = F344	LEW = F344	–		F344 > LEW	LEW > F344	Kruzich and Xi, 2006a
FR1, 6 h; PR	LEW > F344	F344 > LEW	LEW = F344	–			Freeman et al., 2009
FR1, 18 h	LEW = F344			LEW Yes F344 No		–	Picetti et al., 2010
**Amphetamine**							
FR1-FR5, 1 h FR1-FR5, 1 h	LEW > F344 LEW > F344	LEW > F344 LEW > F344	–	–	LEW = F344	LEW = F344	Meyer et al., 2010; Meyer and Bardo, 2015
**Metamphetamine**							
FR1, 2 h	–	LEW > F344	–	–	F344 > LEW	LEW > F344	Kruzich and Xi, 2006b

*For each drug operant schedule (FR, fixed Ratio; PR, Progressive Ratio) and length of session is reported. - data not available*.

It should be emphasized that in food operant SA studies no difference between strains has been detected (Martín et al., 1999; Wilhelm and Mitchell, 2009; Sharp et al., 2011; Chen et al., 2012, but see also Christensen et al., 2009) thus excluding that differences observed between strains in drug SA studies might be due to an inherent difference in reward function in general or due to differences in operant learning.

### Intracranial self-stimulation

Among the paradigms used to study the rewarding effects of drugs the intracranial self-stimulation (ICSS) procedure is also one. This experimental paradigm has been used to study the cerebral circuits mediating reward and was fundamental in the formulation of the reward concept and its application to the current theories of drug consumption and addiction (Olds and Milner, 1954). In this paradigm operant responding is maintained by pulses of electrical brain stimulation delivered through electrodes targeting the medial forebrain bundle at the level of the lateral hypothalamus (Negus and Miller, 2014). Among the different variety of ICSS procedures employed, two of them have been extensively used and validated to study the effects of drugs of abuse: the discrete-trial current-intensity (DT-CI) and the rate-frequency curve shift (R-FCS) (Markou and Koob, 1993). The minimal current needed to promote the ICSS response, is considered a measure of the subject's “reward threshold.” It has been demonstrated that drugs of abuse cause a reduction of the ICSS reward threshold in some brain areas (Kornetsky and Bain, 1992; Wise, 1996) thus increasing the rewarding effect of brain stimulation.

Only limited evidence is available on differences between F344 and LEW strains by using the ICSS paradigm to investigate differences in reward function. While the mean rate-frequency of brain stimulation reward function in the medial forebrain bundle obtained in F344, LEW, and SD rats was not different, administration of 1.0 mg/kg of THC lowered the reward threshold to a greater extent in LEW strain than SD rats, while F344 rats did not appear to be affected by THC (Lepore et al., 1996). This is consistent with microdialysis studies showing that LEW and SD rats are sensitive to the DA releasing properties of THC while F344 rats are not (Chen et al., 1991; Tanda et al., 1997; Cadoni et al., 2008, 2015). In another study using lateral hypothalamic brain stimulation, no difference between strains was observed in the ability of cocaine to potentiate brain stimulation reward (Ranaldi et al., 2001). The observed lack of difference between strains might be due to the drug doses used, given the different effect of low vs. high doses of cocaine on DA transmission in the NAc shell and LEW preference for high doses of cocaine (Cadoni and Di Chiara, 2007; Picetti et al., 2010).

### Impulsivity

Impulsivity is a multifaceted concept mediated by different neural processes, influencing individual behavior in everyday life. It has been defined as the inability to wait, tendency to act without forethought, insensitivity to consequences, preference for immediate over delayed gratification, inability to inhibit inappropriate behavior, tendency to engage in risky behavior, and desire to seek out novel sensations (Reynolds, 2006). Thus, impulsivity has been categorized in two dimensions: *impulsive action* (motor disinhibition) as the inability to inhibit behavior, and *impulsive choice* (impulsive decision making) as preference for immediate over delayed rewards even when the immediate reward is smaller (Jupp and Dalley, 2014). While it is a part of healthy behavior, maladaptive expression of this trait has been associated with a variety of neuropsychiatric disorders. Thus, impulsivity is a core feature of addiction and decades of research have widely demonstrated that impulsivity is antecedent to development of drug addiction (Jupp and Dalley, 2014). The fact that impulsivity is regarded as contributing to the development of addiction, predicting initial drug use, risk for addiction and rate of relapse, as well as response to treatment, together with the fact that drug abuse might affect levels of impulsivity, has unfortunately also been a source of confusion, making unclear whether impulsivity was a cause or a consequence of chronic drug use. The use of animal models has helped to clarify this issue (Winstanley et al., 2010; Jupp et al., 2013).

Studies on impulsivity in these two strains have highlighted how LEW rats are more impulsive than F344 in several experimental paradigms and, as we will see further on, how this behavioral feature can be correlated to other biochemical characteristics of this strain.

In delay discounting tasks, measuring impulsive choice, LEW rats appear to be more impulsive than F344 strain (Anderson and Elcoro, 2007; Madden et al., 2008; Anderson and Diller, 2010; García-Lecumberri et al., 2011; Huskinson and Anderson, 2012; Stein et al., 2012) choosing smaller immediate reinforcers, rather than delayed larger reinforcers and this was true in the case of food reinforcer and morphine infusions.

In a study assessing response impulsivity (or impulsive action), characterized by behavioral disinhibition and associated risky behaviors, LEW rats show more premature responses than F344 rats during the Five Choice Serial Reaction Time Task (5-CSRTT) indicating higher levels of response impulsivity (Hamilton et al., 2014). In autoshaping procedure, LEW rats acquire the autoshaping response faster and performed the autoshaping response at higher rates than F344 rats (Kearns et al., 2006). Autoshaping has been considered to be a form of impulsive behavior (Tomie et al., 1998; Monterosso and Ainslie, 1999) and in particular a form of impulsive action (Winstanley et al., 2004) likely related to impulsive decision-making due to the fundamental role played by CS-US associations in regulating goal-seeking behavior (Winstanley et al., 2005).

The NAc is a key component of the neural processes regulating impulsivity, with afferent and efferent connections and the complex interplay of major neurotransmitter systems within it, making this area a crucial site of behavioral output (Basar et al., 2010). Given the central role played by dopamine (DA) and serotonin (5-HT) in impulsivity, as well as in drug reward, it is conceivable that differences observed between strains might be related to differences in DA and 5-HT transmission in these strains.

Lower DA D2 receptor densities in the striatum and NAc core and lower DA D3 receptor densities in the NAc shell have been observed in LEW as compared with F344 rats (Flores et al., 1998) and therefore would be consistent with a trait of high impulsivity (Dalley et al., 2007; Besson et al., 2010; Moreno et al., 2013). In addition, the lower basal 5-HT levels observed in the NAc and prefrontal cortex (PFCx) of LEW rats (Selim and Bradberry, 1996), as well as the fewer 5-HT receptors in the hippocampus and PFCx (Burnet et al., 1996) have been linked to high levels of impulsivity (see Jupp and Dalley, 2014 for a review).

Although differences between strains in other neurotransmitter systems might concur to determine a high impulsive phenotype (Hayes et al., 2014), as well the reported link between impulsivity and addiction (Jupp and Dalley, 2014), the NAc appears to be a crucial area in regulating not only impulsivity but also drug reward. We will highlight differences between strains not only in basal conditions but also in adaptive changes of DA mesolimbic transmission following repeated drug exposure.

### Anxiety

Somehow contrasting results exist concerning differences in the level of anxiety between the strains. While some studies reported no difference in the level of anxiety between strains (Chaouloff et al., 1995; Rex et al., 1996; Cadoni et al., 2015) others reported an increased anxious state in LEW rats (Kulikov et al., 1997; Ramos et al., 1997; Cohen et al., 2006) that has been related to the hypo-responsivity of their HPA system. These discrepancies might be due to the different anxiety models used that have failed to produce an unique anxiety factor, which also suggests that different experimental models may assess different forms of anxiety (File, 1991; Belzung and Le Pape, 1994). Moreover, previous stressful experiences, novel environment, and handling might have affected animal behavior in anxiety tests (Andrews and File, 1993; Rodgers and Cole, 1993). As an example, handling has been reported to increase open arms entries (decreased anxiety) in plus maze testing, while exposure to a novel environment immediately prior of plus maze testing might enhance or decrease anxiety depending on the strain of the animal.

## Indices of dopamine system functionality

### *In vitro* studies

Studies aimed at identifying differences in DA transmission functionality between LEW and F344 rats have shown that LEW rats, compared with F344, have higher levels of tyrosine hydroxylase (TH, the rate-limiting enzyme for DA synthesis) in the ventral tegmental area (VTA), although having fewer TH positive neurons, thus suggesting higher levels of TH per VTA neuron in this strain (Beitner-Johnson et al., 1991; Harris and Nestler, 1996; Haile et al., 2001). In the NAc, LEW rats show lower levels of TH than F344, while no difference in TH levels or number of positive neurons was detected in the substantia nigra (SN) and in the caudate-putamen (CPu) (Beitner-Johnson et al., 1991; Harris and Nestler, 1996). It is interesting to note that this same pattern of TH expression, i.e., increased levels in VTA and decreased levels in the NAc observed in drug naive LEW vs. F344 rats, has been observed in outbred SD rats after chronic morphine and cocaine (Beitner-Johnson and Nestler, 1991).

Analysis of DA transporter (DAT) levels revealed that LEW rats have lower levels in CPu, NAc, and olfactory tubercle than F344 counterparts (Flores et al., 1998; Haile et al., 2001; Gulley et al., 2007) as well as a lower DAT activity as detected by a slower *in vivo* clearance of locally applied DA in CPu and NAc (Gulley et al., 2007). Interestingly, reduced TH levels in DA terminals in striatum have been found in heroin addicts (Kish et al., 2001), as well reduced DAT levels in methamphetamine addicts (Chang et al., 2007) and in tobacco and marijuana smokers (Leroy et al., 2012).

LEW and F344 rats also differ in DA receptor densities in basal conditions, as well as after exposure to drugs of abuse. LEW rats have been reported to have lower levels of DA D3 receptors in the shell of NAc and olfactory tubercle, lower levels of D2-like receptors in CPu and NAc core compared with F344 rats, while no difference between strains has been observed in D1-like receptor densities in NAc and CPu (Flores et al., 1998). These findings are consistent with lower levels of G_*iα*__1∕2_ and higher levels of adenylate cyclase and cyclic AMP-dependent protein kinase observed in LEW compared with F344 strain (Guitart et al., 1993; Brodkin et al., 1998; Haile et al., 2001).

It is interesting to observe that lower levels of D2 and D3 receptors in the NAc might be consistent with the higher impulsivity of this strain compared with F344 and their vulnerability to drug addiction (Anderson and Woolverton, 2005; Kearns et al., 2006; Madden et al., 2008; Huskinson et al., 2012; Hamilton et al., 2014) given the evidence that lower levels of D2/D3 receptors have been reported to be predictive of trait impulsivity (Dalley et al., 2007; Jupp et al., 2013). Moreover, reduced levels of DA D2 receptors have been detected not only in animals repeatedly exposed to drugs of abuse but also in human addicts (Volkow et al., 1993, 1996, 2001, 2002) while high levels of D2 receptors appear to be protective against alcohol dependence (Thanos et al., 2001; Volkow et al., 2006). In addition several genetic studies in humans have linked D2 receptor gene polymorphisms with addiction to different substances of abuse (Comings and Blum, 2000; Noble, 2000; Le Foll et al., 2009; Agrawal et al., 2012; Gorwood et al., 2012).

In contrast with the above finding, another study showed lower levels of D1-like receptors in the olfactory tubercle and SN and D2-like receptors in the piriform cortex and hippocampal-CA1 in LEW compared with F344 rats, while showing higher D2-like binding in CPu and no difference in the NAc (Sánchez-Cardoso et al., 2009). Lower D1 receptor levels have been also reported in cingulate cortex and higher levels in several areas of hippocampus, thalamus, and SN pars reticulata of LEW when compared with F344 rats (Martín et al., 2003). The lower D1 receptor levels in SN and the higher D2 levels in the CPu have been suggested to reflect a heightened dopaminergic tone in the LEW strain (Sánchez-Cardoso et al., 2009).

This same study also analyzed changes in DA receptor densities after morphine self-administration, showing that in most brain regions analyzed LEW rats displayed a decrease in D1-like binding after morphine self-administration while F344 animals showed an increment, thus suggesting different adaptive changes of DA transmission following morphine self-administration. Additionally, D2 receptors of LEW rats were down regulated after morphine self-administration in the CPu and NAc shell and core while in F344 rats they appeared to be increased in the same areas.

Other interesting differences between the two strains are the lower levels of cholecystokinin (CCK) immunoreactivity in the VTA and the lower extraneuronal levels of CCK_8_ and CCK_2_ receptors detected in the NAc of LEW as compared with F344 strain (Harris and Nestler, 1996; Noble et al., 2012). In fact, the neuropeptide CCK, co-localized with DA in a subpopulation of VTA neurons, but also in SN pars compacta (Hökfelt et al., 1980; Seroogy et al., 1989), has been hypothesized to be involved in modulating the emotional and motivational functions of morphine (Faris et al., 1983) through an action on DA mesolimbic system (Wang et al., 1985; Voigt et al., 1986; Ladurelle et al., 1993). Thus, low levels of CCK in the NAc has been hypothesized to increase the reinforcing effects of drugs of abuse (Rotzinger and Vaccarino, 2003; Noble et al., 2012).

### *In vivo* studies

Given the pivotal role of mesolimbic DA transmission in the motivational and rewarding properties of drugs of abuse, extensive research has been focused on investigating differences in mesolimbic DA function and responsiveness to drugs. As reported above, LEW and F344 rats differ in several indices of DA transmission. Therefore, it was predictable that they would have shown differences in DA transmission as recorded by *in vivo* techniques. Electrophysiological and brain microdialysis studies have been useful to unveil these differences.

With regards to electrophysiology of DA neurons, only two studies have investigated differences between F344 and LEW strains. Minabe et al. (1995), by using *in vivo* extracellular recording techniques, showed that LEW rats have a significantly lower number of spontaneously active DA neurons in the VTA and SN pars compacta compared with F344 rats, consistent with their lower number of TH positive neurons (Harris and Nestler, 1996). Spontaneously active DA neurons in the VTA of LEW rats display significantly greater % events as burst and % of cells exhibiting burst firing pattern compared with F344 strain, although no difference was observed in the % of spikes in bursts as well as in the variation coefficient (Minabe et al., 1995).

Early microdialysis studies have led to contrasting results, likely due to differences between studies in targeting specific and defined areas.

Thus, while Camp et al. (1994) reported greater increases in extracellular DA in the ventral striatum of LEW rats after different doses of *methamphetamine* and *cocaine*, Strecker et al. (1995) did not find significant differences between LEW and F344 rats in the increase of extracellular DA in the NAc following different doses of cocaine. Fernandez et al. (2003a) did not observe differences in DA increase after a low dose of *methylendioxymethamphetamine* (MDMA), while reporting a greater DA increase in the NAc shell of F344 strain with a high dose. These early studies did not report any significant difference in basal DA levels between strains, but showed markedly lower levels of DOPAC and HVA (DA metabolites) in LEW compared with F344 rats (Camp et al., 1994; Strecker et al., 1995), as well as higher plasma and brain levels (two to three-fold) of methamphetamine and cocaine in LEW strain (Camp et al., 1994 but see also Kosten et al., 1997), thus suggesting differences not only in DA clearance but also in drug pharmacokinetics.

Another study aimed to investigate the effect of *ethanol* on mesolimbic DA transmission did not show differences between strains after 0.5 and 2.0 g/kg i.p., while administration of 1.0 g/kg increased DA only in F344 strain (Mocsary and Bradberry, 1996). Sziraki et al. (2001) while reporting a greater DA increase in the NAc shell of LEW rats after *nicotine* and a greater increase after *amphetamine* in F344 strain, also observed basal DA levels three times higher in F344 vs. LEW rats.

Given these somewhat contrasting results we performed a systematic study to evaluate differences between strains in mesolimbic DA transmission responsiveness to different classes of drugs of abuse in the NAc shell and core. In fact, it is today well recognized that these two subdivision of the NAc are not only anatomically and functionally distinct but also play different roles in mediating locomotor, motivational and reinforcing properties of drugs of abuse. DA in the NAc shell is involved in reward encoding, incentive learning and thus mediating motivational valence. On the other hand, DA in the NAc core appears to be involved in conditioned responding, based on stimulus-outcome association, and thus in Pavlovian cue encoding and in goal directed actions, playing a role in selection between actions of differing value (Di Chiara, 2002; Di Chiara et al., 2004; Ikemoto, 2007; Bromberg-Martin et al., 2010; Saddoris et al., 2013).

Our studies have shown that although basal DA levels in the shell and core of NAc were not significantly different between strains, LEW rats display in general a higher DA responsiveness than F344 rats to morphine, cocaine, amphetamine, nicotine and (THC), although some exceptions were observed depending on the drug dose and the area analyzed (shell vs. core).

Following *cocaine* challenge, a greater response in the NAc core at doses of 5 and 10 mg/kg was observed, while a greater response in the NAc shell was obtained at 20 mg/kg in LEW compared with F344 rats (Cadoni and Di Chiara, 2007).

Following challenge with *amphetamine*, an increased DA release in the NAc core of LEW rats was observed at any drug dose tested (0.25, 0.5, and 1.0 mg/kg) while in the NAc shell DA response was greater in LEW after the lower dose and greater in F344 rats after the higher dose.

*Morphine* administration elicited a greater DA response in the NAc shell of LEW rats except at the lowest dose (1.0 mg/kg) and in the core at the lowest and highest dose but not after the intermediate dose (Cadoni and Di Chiara, 2007).

*Nicotine* administration elicited higher DA increase in the NAc shell of LEW rats except at the highest dose tested and at all doses in the NAc core, while an increase in this area was not observed in F344 strain (Cadoni et al., 2009).

Moreover, *THC* administration elicited a selective DA increase in the NAc shell of LEW rats while it did not affect DA transmission in F344 rats (Cadoni et al., 2015), thus in agreement with previous studies showing that LEW rats are the most sensitive, among different rat strains, to the rewarding and DA increasing properties of THC (Chen et al., 1991; Lepore et al., 1996).

The results above reported are consistent with previous CPP and self-administration studies showing a greater morphine, cocaine and nicotine CPP and SA in LEW as compared with F344 rats, with the only exception being amphetamine, which induces a greater CPP in F344 strain (Stöhr et al., 1998). We suggested that the reinforcing properties of the drug in CPP paradigms might depend on the relative ratio (shell vs. core) of DA transmission stimulation rather than on the absolute DA increase obtained in the shell or core of NAc (Cadoni and Di Chiara, 2007), given the different roles played by DA in the two subdivisions of the NAc in mediating drug reinforcing properties and motivated behavior (Fenu et al., 2006; Spina et al., 2006).

Other factors to be considered in evaluating drug effects on mesolimbic DA transmission are differences of these two strains in other neurotransmitter systems (serotonin, glutamate, GABA, endocannabinoid, opioid) as well as their different HPA activity, which play an important role in the final effect of the drug on DA transmission, particularly of psychostimulant drugs.

Apart from being differently sensitive to the DA releasing effects of different drugs of abuse, LEW rats compared with their F344 counterparts appear to develop different adaptive changes in mesolimbic DA transmission. Thus, while adult and adolescent SD (Cadoni and Di Chiara, 1999, 2000; Cadoni et al., 2000, 2008) and F344 rats (Cadoni et al., 2015) show a reduced or unchanged DA transmission responsiveness in the NAc shell and an increase in the NAc core, after repeated non-contingent exposure to different drugs of abuse, LEW rats display an increased responsiveness of DA transmission in the NAc shell after adolescent THC exposure (Cadoni et al., 2015). Sensitization of DA transmission in the NAc core is not always observed, but that may be due to the fact that LEW rats have already an increased DA responsiveness in the NAc core at basal conditions (Cadoni and Di Chiara, 2007; Cadoni et al., 2009). These data are particularly intriguing because they suggest that LEW rats, even after repeated exposure to the drug, maintain an unchanged or even enhanced motivational valence, incentive arousal or reward encoding of the drug stimuli (Di Chiara and Bassareo, 2007; Bromberg-Martin et al., 2010; Saddoris et al., 2013).

## Vulnerability to stress

Copying with aversive situations is the result of complex information processing and defensive response expression. The hypothalamic-pituitary-adrenocortical (HPA) axis, activated by stressful events leading to the release of corticosteroids (cortisol in humans and corticosterone in rodents), is a key structure of the stress reaction. Activation of the HPA axis gives rise to a cascade of events involving the release of corticotropin releasing factor (CRF) from the paraventricular nucleus (PVN) of the hypothalamus that leads to enhanced secretion of adrenocorticotropic hormone (ACTH) from the pituitary. ACTH in the general circulation stimulates the secretion of corticosteroids in the adrenal cortex.

In the brain corticosteroids bind to two types of receptors: mineralocorticoid receptor (MR or Type I) and glucocorticoid receptor (GR or Type II) (Gallagher et al., 2009). GRs are widely distributed in the brain in cortical as well in limbic areas, while MRs have been detected in hippocampal, thalamic, and hypothalamic areas. MRs have greater affinity for corticosterone (CORT) thus being almost saturated at basal levels, while GR occupancy increases during circadian peak or stressful stimuli (Reul and de Kloet, 1985). An interesting observation for our discussion is the localization of GRs on DA neurons of VTA and SN (Hensleigh and Pritchard, 2013) thus being able to directly affect DA neuron functionality (Marinelli et al., 1994, 1998; Piazza and Le Moal, 1996; Piazza et al., 1996; Graf et al., 2013), while DA transmission exerts a negative control on corticosteroids receptors (Casolini et al., 1993).

Extensive research has been made on differences in the HPA responsiveness of these two strains also because they are used as a model of different susceptibility to inflammatory diseases, given the link between HPA responsiveness and immune function (Elenkov et al., 2008; Macho et al., 2008; Silverman and Sternberg, 2012).

### Stress and addiction

Substantial evidence shows that HPA axis and corticosteroids are involved in the process of addiction (Sarnyai et al., 2001; Koob, 2009; Vinson and Brennan, 2013) and that substances of abuse are able to activate HPA axis by themselves (Armario, 2010). Thus, the difference in HPA axis responsiveness between these two strains (Kosten and Ambrosio, 2002) offers a unique opportunity to further investigate the role of vulnerability to stress in relation to addiction vulnerability.

Although it is well recognized that the HPA axis and corticosteroids are involved in drug sensitivity and in the process of addiction, what is less clear is whether individuals at risk of developing addiction have a heightened or a blunted response of HPA to stressful stimuli, thus being less or more sensitive to relapse after stressful stimuli or being less or more successful at quitting drug abuse (Lovallo, 2006; Schumann, 2006; Koob and Kreek, 2007; Fox et al., 2009; Paris et al., 2010; McKee et al., 2011; Sinha, 2011; van Leeuwen et al., 2011; Evans et al., 2012; Vinson and Brennan, 2013; Goeders et al., 2014). On the other hand in pre-clinical studies while it was reported that locomotor response to stress, anxious phenotype and high corticosterone (CORT) levels were related to heightened drug self-administration, later studies demonstrated that these factors were not predictive of development of addiction-like behavior (see Piazza and Deroche-Gamonet, 2013 for a review).

### HPA axis responsiveness in F344 and LEW rats

Under basal conditions no difference was observed between strains in CRF mRNA in PVN, ACTH and CORT serum levels, and in adrenal weight, as well as in hippocampal GR and MR mRNA (Sternberg et al., 1992; Gómez et al., 1996; Grota et al., 1997; Baumann et al., 2000; Ergang et al., 2014, 2015). At variance with the above studies, other authors have found reduced CORT serum levels, higher GR receptor levels in thymus, lower MR levels in hippocampus and pituitary, and significantly lower levels of corticosteroid-binding globulin (CBG) in LEW compared with F344 and SD strain (Dhabhar et al., 1993; Smith et al., 1994; Macho et al., 2008; Table 2).

**Table 2:** **Differences between LEW and F344 strains in HPA axis activity in different conditions**.

	**CRF**	**ACTH**	**CORT**	**GR levels**	**GR activation**	**MR levels**	**11HSD1**	**CBG**	**References**
Basal conditions	LEW=F344	LEW=F344	LEW=F344	LEW=F344	–	LEW=F344	–	–	Sternberg et al., 1992; Gómez et al., 1996; Grota et al., 1997; Baumann et al., 2000
								–	
							LEW=F344 (dCA1,CA2)		Ergang et al., 2014
			LEW < F344				LEW>F344 (PVN, pituitary)		Ergang et al., 2015
				LEW>F344 (thymus)			LEW<F344 (PfCx,vCA1,CA3)	LEW<F344	Dhabhar et al., 1993; Macho et al., 2008
						LEW<F344 (hippocampus, pituitary)	–		Smith et al., 1994
Circadian variation			♀ LEW Yes						Griffin and Whitacre, 1991; Windle et al., 1998 Griffin and Whitacre, 1991; Dhabhar et al., 1993; Gómez et al., 1996
			♀ F344 No						
			♀ LEW No						
			♁ F344 Yes						
Stress	LEW<F344	LEW<F344	LEW<F344			–	–	–	Sternberg et al., 1992; Dhabhar et al., 1993; Rivest and Rivier, 1994; Armario et al., 1995; Shurin et al., 1995; Stöhr et al., 2000; Moncek et al., 2001; Ergang et al., 2014
	LEW=F344								Rivest and Rivier, 1994
			LEW>F344 (adrenal cortex)						Moncek et al., 2001
				↓LEW=F344 (dorsal CA1 hippocampus)	LEW<F344 (brain and immune tissue)				Dhabhar et al., 1995
	LEW>F344 (amygdala)						↑F344 ↔LEW (ventral CA1 hippocampus)		Ergang et al., 2015
Prolonged stress exposure	–	LEW < F344	LEW < F344	–	–	–	–	–	Dhabhar et al., 1997
Chronic stress	↑ LEW=F344	LEW > F344	LEW < F344 LEW < F344	↓ LEW=F344 (hippocampus)	–	↓ LEW=F344 (hippocampus)	–	–	Gómez et al., 1996 Dhabhar et al., 1997
Nicotine	–	–	LEW < F344	–	–	–	–	–	Grota et al., 1997
Morphine	–	–	LEW < F344	–	–	–	–	–	Baumann et al., 2000
Cocaine	LEW < F344	LEW > F344	LEW < F344 LEW > F344 (as % of basal)	–	–	–	–	–	Simar et al., 1996

*Data of CORT and ACTH refer to serum levels, CRF refers to mRNA expression in paraventricular nucleus (PVN) of the hypothalamus (except for data following cocaine administration) as well as GR and MR data refer to mRNA levels in the areas indicated. Abbreviations: CRF corticotrophin releasing factor, ACTH adrenocorticotropic hormone, CORT corticosterone, GRs glucocorticoid receptors, MRs mineralocorticoid receptors, 11HSD1 11β-hydroxysteroid dehydrogenase type 1, CBG corticosteroid-binding globulin. Symbols used: ↑ up-regulation; ↓ down-regulation; ↔ no change; - data not available*.

Different pulsatile characteristics of the HPA have been observed between strains depending on sex, with female LEW rats showing circadian variation of CORT plasma concentrations which was not present in female F344 rats (Griffin and Whitacre, 1991; Windle et al., 1998), thus affecting CORT response to stress stimuli depending on the phase (rising or falling) of the basal pulse. While male F344 rats show significantly higher diurnal, as well as stress induced, CORT levels compared with LEW and SD rats, returning rapidly to basal levels after stress differently from LEW, male LEW rats show a blunted CORT circadian variation compared with F344 (Griffin and Whitacre, 1991; Dhabhar et al., 1993; Gómez et al., 1996). This finding is of particular importance in experimental protocols conducted at different time of the day.

Several studies (Table 2) have shown differences between strains in HPA axis activation following different kinds of acute stress. Reduced CRF mRNA expression after restrain stress in the PVN has been reported in LEW as compared with F344 rats (Sternberg et al., 1992), or unchanged post footshock stress (Rivest and Rivier, 1994). Plasma ACTH and CORT levels in response to different kinds of stress are reduced in LEW compared with F344 rats (Sternberg et al., 1992; Dhabhar et al., 1993; Rivest and Rivier, 1994; Shurin et al., 1995; Stöhr et al., 2000; Moncek et al., 2001; Ergang et al., 2014) but also compared with other strains of rats (Armario et al., 1995; Gómez et al., 1998; Deutsch-Feldman et al., 2015). In this regard, SD rats have been reported to have intermediate responses between F344 and LEW strains (Sternberg et al., 1992; Dhabhar et al., 1993). Another study using acute immobilization stress while confirming the above findings about lower ACTH and CORT levels in LEW compared with F344 strain, reported significantly higher CORT concentration in the adrenal cortex of LEW than F344 rats (Moncek et al., 2001).

Interestingly, in a study aimed to evaluate the influence of handling and injection protocols on HPA response, differences in CORT blood concentrations have been observed between LEW and SD strain (Deutsch-Feldman et al., 2015). While CORT concentrations in SD rats decreased after continued handling, in LEW rats they remained constant. The fact that this attenuation does not extend to the ACTH response suggests an effect at the adrenal level. This finding underscores the importance of minimizing stress associated with experimental procedures that might have different impacts on the rat strain used, thus sometimes leading to inconsistent results.

Experimental manipulation of endogenous negative glucocorticoid feedback by dexamethasone administration showed a greater reduction of ACTH and CORT plasma levels in response to acute stress (tail shock) in LEW as compared with F344 rats, while pharmacological adrenalectomy causes an increase in ACTH levels in F344 but not in LEW rats following the same acute stress (Gómez et al., 1998).

After chronic immobilization stress a similar up-regulation of CRF mRNA expression in the hypothalamic PVN was observed in the two strains, while ACTH serum levels were increased in LEW but not in F344 rats; vice versa basal CORT serum levels were increased in F344 but not in LEW (Gómez et al., 1996). Moreover, F344 rats did not show habituation to prolonged stress exposure of the ACTH and CORT response compared with LEW as well as SD rats (Dhabhar et al., 1997).

A short-term variable stress protocol also stimulates the expression of amygdalar CRF only in LEW and UNC2/UCN3 (urocortins 2/3) only in F344 rats (Ergang et al., 2015). The observed CRF increase in amygdala following stress in LEW, but not in F344 rats, is of particular interest since it has been involved in addiction development as well in relapse mechanisms (Koob, 2015; Zorrilla et al., 2014).

It is also worth noting the finding that prenatal stress differentially affects expression and processing of hippocampal brain-derived neurotrophic factor (BDNF) in the offsprings of the two strains. Thus, LEW (but also SD) rats show a decreased BDNF mRNA expression immediately after stress instead of showing increased levels, and also a decreased amount of available proBDNF and mature BDNF protein suggesting a higher vulnerability to prenatal stress compared with F344 strain (Neeley et al., 2011).

Differences between strains are not limited to the main players of HPA activity, but extend to other peptides playing key roles in the regulation of HPA activity, in basal conditions as well as following stress. Thus, LEW rats constitutively have higher levels of 11β-hydroxysteroid dehydrogenase type 1 (11HSD1), an enzyme that regulates the conversion of glucorticoids from inactive to active form, in the PVN and pituitary, while having lower levels in PfCx, ventral CA1, CA3 areas of hippocampus, and adrenal cortex (Ergang et al., 2015). Stress up-regulates 11HSD1 in the prefrontal cortex and lateral amygdala in LEW rats, whereas in F344 11HSD1 is up-regulated in central amygdala, hippocampal CA2, and ventral CA1, while no effect was detected in the PVN, pituitary gland and adrenal cortex of both strains (Ergang et al., 2015).

Differential activation of GRs in neural and immune tissue during stress was observed, with F344 strain exhibiting the highest receptor activation in brain tissue (hippocampus, hypothalamus, cortex, and pituitary) while LEW rats exhibiting the lowest magnitude of GR activation in immune tissues (Dhabhar et al., 1995). A similar down-regulation of GR mRNA following chronic stress or variable stress protocol in the dorsal but not ventral CA1 area of hippocampus was observed in the two strains (Gómez et al., 1996; Ergang et al., 2014).

Differential reactivity of HPA in the two strains is not limited to stress exposure but also extends to exposure to drugs of abuse. Thus, LEW compared with F344 rats show a reduced CORT response to nicotine (Grota et al., 1997) and morphine challenge (Baumann et al., 2000). Following administration of different doses of cocaine, LEW rats display reduced CORT response compared with F344 strain, but show higher response when expressed as percent of change, while plasma ACTH concentrations, as well as percentage of control response, were dramatically increased in LEW rats after 40 mg/kg of cocaine (Simar et al., 1996). Interestingly increased levels of CORT and ACTH were observed in F344 rats during the first 24 h of withdrawal after an extended access paradigm of cocaine SA, while LEW rats did not show any change although they escalated their cocaine intake during this experimental procedure (Picetti et al., 2010).

As mentioned at the beginning of this section, HPA activity and immune function are strictly linked (Silverman and Sternberg, 2012). Given that the neuroimmune system appears to be involved not only in aversive and stress response but also in affective disorders and drug addiction (Crews et al., 2011; Cui et al., 2014) the above reported vulnerability of LEW rats to develop not only drug addiction but also neuroinflammatory diseases (Elenkov et al., 2008) offers a unique opportunity to further investigate the role of neuroinflammation in the pathophysiology of addiction (Cui et al., 2014; Ray et al., 2014; Rodrigues et al., 2014).

In conclusion, although LEW rats appear to have a hypo-responsive HPA axis in certain conditions it is not clear from the literature (Armario et al., 1995; Stöhr et al., 2000; Grakalic et al., 2006; Kosten and Miserendino, 2012) if this phenotype might be associated with a lower or higher vulnerability to stress and therefore being a factor of vulnerability to addiction. The fact that, in experimental models of post-traumatic stress disorders (PTSD), LEW rats appear to be more susceptible than F344 rats to PTSD-like responses, suggests that blunted HPA response might be a factor of vulnerability to stressful events and recovery from them (Cohen et al., 2006). Thus, it might be hypothesized, on the basis of previous considerations, that a reduced HPA activity might be a vulnerability factor given the reduced ability of the LEW strain to cope with adverse or stressful events. Further studies are clearly needed to clarify this issue.

## Differences in other neurotransmitter systems

Although DA system is a key player in the processing of reward and motivation and thus in drug addiction, differences in other neurotransmitter systems may concur to shape an addiction prone phenotype.

One of these systems playing a critical role is the *endocannabinoid system* (Solinas et al., 2008; Melis and Pistis, 2012; Panagis et al., 2014; Wenzel and Cheer, 2014). Early studies suggested that LEW rats are more sensitive to the rewarding effect of THC thus leading us to hypothesize the existence of differences between strains in the endocannabinoid system (Chen et al., 1991; Lepore et al., 1996; Cadoni et al., 2015). However, only few studies have investigated differences between the two strains in indices of endocannabinoid system functionality. An analysis of several brain areas showed lower cannabinoid (CB) receptor binding in the globus pallidus (GP) and a higher cannabinoid receptor 1 (CNR1) gene expression in the PfCx of LEW compared with F344 rats (Coria et al., 2014). In the hippocampus, LEW rats show lower expression of CB1 receptors and higher CB2 receptors than F344 rats (Rivera et al., 2013). Moreover, F344 rats have been reported having reduced levels of CB1 receptors and FAAH in the hippocampus compared with Wistar rats (Brand et al., 2012).

As regards levels of enzymes mediating synthesis and degradation of endocannabinoids, LEW strain displays increased expression of the genes encoding fatty acid amide hydrolase (FAAH), monoacylglicerol lipase (MAGL) in the PfCx and in the case of FAAH also in the NAc (Coria et al., 2014). Moreover, the N-acyl phosphatidylethanolamine phospholipase D (NAPE-PLD)/FAAH ratio is lower in the PfCx, NAc, and CA3 hippocampal field of LEW as compared with F344 rats, suggesting higher anandamide levels in F344 strain (Rivera et al., 2013; Coria et al., 2014).

Given the pivotal role of the endocannabinoid system in brain development (Harkany et al., 2008), differences in levels of synthesis and degrading enzymes, and in receptor density and distribution, might affect adolescence vulnerability of these strains to various drugs of abuse. Indeed, we have reported that adolescent exposure to THC induces markedly different effects on reward function in the two strains (Di Chiara et al., 2013; Cadoni et al., 2015). Differences in the endocannabinoid system acquire a particular relevance in the light of recent evidence on the control of midbrain DA neurons activity by endocannabinoids in the VTA (Barrot et al., 2012; Melis and Pistis, 2012; Wang and Lupica, 2014). It is worth noting that in human genetic studies genetic variation in the gene coding for the CB1 receptor (CNR1), as well as for FAAH and MAGL (MGLL) has been reported to be associated not only with cannabis dependence, but also with substance use disorder in general and impulsive personality traits (Agrawal and Lynskey, 2009; Proudnikov et al., 2010; Wang et al., 2011; Bidwell et al., 2013; Clarke et al., 2013; Demers et al., 2014).

With regard to differences between strains in *glutamate (GLU)* and *GABA transmission* it has been reported that LEW rats have lower basal levels of Glu in the PfCx and NAc shell and core and lower GABA levels in the NAc core (Selim and Bradberry, 1996; Miguéns et al., 2013).

Cocaine SA has been reported to alter long term potentiation (LTP) depotentiation in the hippocampus of LEW, but not F344 rats (Miguéns et al., 2011) suggesting that long-term exposure to cocaine impairs the synaptic plasticity that supports learning. Moreover, these neurotransmitter systems appear to respond differently after cocaine priming reinstatement in a SA paradigm with LEW rats showing sharp increase in GLU levels and F344 rats showing an increase in GABA levels (Miguéns et al., 2013). This result has been hypothesized to be at the basis of the greater vulnerability of LEW rats to cocaine reinstatement.

Another study showed that long term depression (LTD) induced by GLU agonist NMDA is partially abolished in hippocampal slices of LEW rats exposed during adolescence to cocaine, while in F344 rats hippocampal NMDA-LTD is partially inhibited independently of cocaine treatment, suggesting lack of plasticity of this strain which could be related to its poor performance in spatial memory tasks (Fole et al., 2015). It would be interesting to investigate differences between strains in LTD in the NAc given that impaired LTD has been suggested to be related to transition to cocaine addiction in an animal model (Kasanetz et al., 2010).

Moreover, LEW rats have been reported to have significantly higher benzodiazepine binding sites (Bmax) in hypothalamic preparations compared with F344 rats but show no difference in binding affinities (Kd). These differences appear only partially related to strain differences in CORT levels (Smith et al., 1992), and suggest a different GABA functionality in this area.

Several lines of evidence indicate that LEW strain has a reduced *serotonin (5-HT) neurotransmission* function. Thus, a reduced activity of tryptophan hydroxylase (the rate-limiting enzyme in 5-HT biosynthesis) in hippocampus (Chaouloff et al., 1995) and higher hippocampal extracellular 5-HT basal levels has been observed in LEW vs. F344 rats (Fernandez et al., 2003b). In the NAc LEW rats display lower basal 5-HT levels compared with F344 counterparts but increased 5-HT levels (but also GLU levels) following ethanol challenge, responses that were absent in the F344 strain (Selim and Bradberry, 1996). Female and male LEW rats have been shown to have lower levels of serotonin transporter (5-HTT) gene expression in midbrain and hippocampal preparations and lower hippocampal 5-HT reuptake sites compared with F344 counterparts, consistent with the higher 5-HT levels in this area (Fernandez et al., 2003b). LEW have significantly fewer 5-HTR binding sites in hippocampal and frontal cortical regions and less 5-HT1A receptor mRNA compared with SD and F344 rats (Burnet et al., 1992; Chaouloff et al., 1995).

While a reduced 5-HT tone might be the basis of the greater impulsivity of LEW rats, nonetheless differences in 5-HT system function might affect reward processing given the role of this system in reward function in general and in modulating DA transmission in particular (Alex and Pehek, 2007; Kranz et al., 2010).

LEW rats also appear to have a reduced *opioid function*. Thus, LEW rats display lower basal dynorphin peptide levels in SN, CPu, and VTA (but not dynorphin B) and in the pituitary gland. Leu-enkephalinArg6 levels were also lower in these structures and in the NAc, with the only exception of the CPu where levels were higher in LEW compared with F344 rats (Nylander et al., 1995).

Basal proenkephalin (PENK) mRNA expression was also found to be reduced in CPu and NAc shell of LEW rats (Martín et al., 1999; Sánchez-Cardoso et al., 2007).

Adaptive changes following chronic morphine were different in the two strains with only F344 rats showing increased dynorphin A levels in NAc and VTA and LEW showing a decrease in VTA and hippocampus (Nylander et al., 1995). Additionally, only LEW rats showed an increase of dynorphin peptide levels during withdrawal. Leu- and Met-Enkephalin peptide levels were increased in F344 rats after chronic morphine while not affected in LEW rats. These differences acquire a particular significance in the light of the different actions of these peptides (dynorphin A and B act on κ opioid receptors while enkephalin peptides act on δ and μ receptors) and considering the known effect of these receptor activations on DA transmission (Di Chiara and Imperato, 1988; Spanagel et al., 1990; Devine et al., 1993).

LEW rats self-administering morphine showed a decrease of PENK mRNA content in every area, which recovered to normal levels during extinction, except for the NAc shell where no change was apparent (Sánchez-Cardoso et al., 2007).

LEW rats in basal conditions display lower μ opioid receptor (MOR) levels not only in cerebral cortex and spinal cord (Herradón et al., 2003) but also in several other brain areas (Sánchez-Cardoso et al., 2007) which persisted after morphine self-administration in some of them (including CPu, NAc, amygdala, SN pars reticulata) but not in others (Sánchez-Cardoso et al., 2007).

In morphine self-administering rats opposite effects were observed in the two strains with LEW rats showing increased binding levels to MORs in CA1 and CA2 fields of the hippocampus while F344 rats showed a decrease. An increased binding to MORs was also observed in primary and secondary motor cortex, M1 and M2, NAc shell and core, VTA and other nuclei (Sánchez-Cardoso et al., 2007). The most common pattern of changes in MORs during extinction training was a decrease until day 7 followed by recovery until day 15, which was more prominent in F344 rats. This increased MOR binding observed in LEW strain might be consistent with higher scores of craving-like reactions observed in LEW rats after repeated exposure to heroin (Cadoni et al., 2015). It is interesting to note in this regard that in the brains of heroin users, deceased because of overdose, upregulation of MOR G-protein coupling has been observed in the paranigral nucleus of VTA (Horvath et al., 2007). Increased MOR binding was also observed in cortical areas of cocaine users, which correlated with cocaine craving and predicted treatment outcome (Zubieta et al., 1996; Gorelick et al., 2008; Ghitza et al., 2010).

Moreover, functionality of MORs appears to be different between strains and in basal conditions LEW rats show higher functionality of these receptors than F344 rats in NAc core and cingulate cortex, area 1 (Cg1), (Sánchez-Cardoso et al., 2007), which is reversed after morphine SA in the lateral amygdala (La). At the end of extinction period strain differences were preserved only in the NAc shell, La and paraventricular thalamic nucleus suggesting that LEW strain has a lower functional activation of μ opioid receptors.

In summary, the endogenous opioid system seems to be down-regulated in the LEW strain both from a structural point of view (number of receptor binding sites and PENK gene expression) but also from a functional one, showing different adaptive changes in opioid receptors and PENK gene expression.

Only limited evidence exists on the *noradrenergic system* function in these two strains. Herradón et al. (2006) did not find differences in mRNA levels for α_2A_ and *a*_2C_ adrenoreceptors in any of the areas analyzed (hypothalamus, hippocampus, striatum and cortex) but they found lower levels of α_2B_ adrenoreceptors transcripts in the hippocampus and higher levels in hypothalamus of F344 rats. TH gene expression was found to be higher in the hippocampus and CPu of F344 as compared with LEW rats, but a significant increase of the protein levels was detected only in the case of hippocampus. Given the crucial role of the hippocampus in learning and memory and thus in drug addiction, the reported differences between strains in this area involving not only noradrenergic transmission but other systems (see above) as well, might play a role in the observed strain differences in drug seeking and taking behavior.

## Structural plasticity and differences in genes expression

Experience dependent changes in behavior and psychological functions such as those related to learning and memory processes and thus, also those occurring during development of addiction, are the result of changes in neuronal plasticity associated with reorganization of synaptic connections (structural plasticity) in relevant brain circuits (Nestler, 2001; Lamprecht and LeDoux, 2004; Robinson and Kolb, 2004; Spiga et al., 2014). Several studies have investigated the effects of contingent and non-contingent exposure to drugs of abuse on neuron morphology such as spine density and dendritic branching of pyramidal cortical neurons and NAc medium spiny neurons. Indeed, exposure to amphetamine, cocaine, nicotine, and morphine induces persistent changes in dendrites and dendritic spines of neurons not only in brain regions involved in reward processing but also in decision making and inhibitory control of behavior (Robinson and Kolb, 2004; Spiga et al., 2014). This synaptic reorganization, which alters the functioning of these circuits, might explain the persistence of some behavioral aspects of addiction such as craving.

Few studies have been performed investigating different structural plasticity in F344 and LEW strains.

Two studies comparing the effects of morphine SA on basal dendritic arbors of layer III pyramidal neurons in both prelimbic (Plc) and motor cortex (Mc) found differences between LEW and F344 strains not only after morphine SA but also in saline control animals. In basal conditions (animals self-administering saline), while no significant differences in pyramidal neuron morphology were observed between Mc and Plc of F344 rats (Ballesteros-Yáñez et al., 2007), LEW rats showed differences between areas, having neurons with a smaller and less branched dendritic arbor, and a higher density of spines in the Plc compared to those in the Mc. Strain comparison in each area showed that in the Plc LEW rats have a higher number and density of spines, and in the Mc have higher values in several morphological parameters (branching pattern, dendritic length, length per distance, length per order, and number of spine) compared to F344 (Ballesteros-Yanez et al., 2008).

Following morphine self-administration, Plc pyramidal neurons of LEW rats had larger and longer dendritic arbors while in the Mc there was a reduction in the size and branching complexity of the dendritic arbors of pyramidal neurons. Spine density was increased in both cortical regions following morphine exposure (Ballesteros-Yáñez et al., 2007). The same morphine exposure in F344 rats did not induce any change in the structure of the dendritic arbors or in the spine density of pyramidal neurons in either cortical area (Ballesteros-Yanez et al., 2008). The above results following morphine SA would suggest that F344 rats receive fewer excitatory inputs since the majority of spines establish at least one excitatory GLU synapse (Arellano et al., 2007).

Moreover, significant strain differences in the structure of apical dendrites of hippocampal CA1 pyramidal neurons were observed in saline and cocaine exposed animals, with LEW strain showing more branched and complex CA1 apical dendrites than F344, although spine density in saline-LEW rats was lower than that of F344 rats. As previously reported in morphine SA studies, cocaine SA also produces different structural changes in hippocampal CA1 pyramidal neurons in the two strains. While cocaine SA increases spine density in apical dendrites of CA1 pyramidal cells of LEW rats, no such change was observed in F344 rats (Miguéns et al., 2013). Cocaine SA had no effect on any of the branch complexity parameters analyzed (Miguéns et al., 2013). The reduced dendritic arborization observed in F344 rats could be related to the different ability of this strain, compared with LEW, in acquiring cocaine SA (Kosten et al., 1997; Freeman et al., 2009). On the other hand, the effect of cocaine in increasing spine density in LEW, but not in F344 rats, might contribute to increased drug intake observed in this strain in extended access paradigms (Picetti et al., 2010). An in-depth 3D analysis of CA1 dendrites and dendritic spines in these two strains prior to cocaine SA show that LEW rats have significantly larger dendritic diameters but lower spine density compared with F344 strain. After cocaine SA, proximal dendritic volume, dendritic surface area, and spine density were increased in LEW strain, where also an increased percentage of larger spines was observed. In contrast, F344 rats showed decreased spine head volume after cocaine SA (Selvas et al., 2015).

In studies on hippocampal CA1 neuronal morphology chronic cocaine administration during a spatial learning task increased spine density in both strains, but to a greater extent in F344 rats, while in saline treated animals F344 rats showed an increased spine density following the same task (Fole et al., 2011). It has been suggested that the increase in spine number could be related with learning deficits and this might account for the lower capacity of F344 rats in learning the task (Fole et al., 2011). Thus, strain differences in hippocampal neuron morphology might represent the anatomical substrate for differential ability in performing spatial learning tasks.

All these observations might account for differences in memory processing of drug reward cues thus contributing to the different vulnerability to addiction of these two strains.

Few studies have investigated differences in gene expression between these two strains. One, investigating genes induced and repressed in frontal cortex and NAc, found only a limited number of genes that were differentially expressed in the two strains (Higuera-Matas et al., 2011). The genes that were induced in the LEW strain were related to oxygen transport, neurotransmitter processing, and fatty acid metabolism while genes that were repressed in LEW rats were involved in physiological functions such as drug and proton transport, oligodendrocyte survival and lipid catabolism. Another study aimed at evaluating differences in basal gene expression in NAc GABA neurons projecting to ventral pallidum and found 322 transcripts that differed between these two strains (Sharp et al., 2011). The observation of a significant up-regulation in LEW rats of some genes (Mint-1, Cask, CamkIIδ, Ncam1, Vsnl1, Hpcal1, and Car8) involved in cellular signaling and synaptic plasticity, suggests that these gene transcripts may contribute to altered function of NAc GABA neurons that might predispose LEW rats to self-administer nicotine (Sharp et al., 2011), but also other drugs. It is noteworthy that increased Ncam1 (neural cell adhesion molecule 1) expression appears to reduce D2 receptor levels (Xiao et al., 2009), which inhibit NAc GABA neuron activity. Moreover, Vsnl1 (Visinin-like 1) interacts directly with the alpha4 subunit of the most abundant nicotinic cholinergic receptor alpha4beta2, increasing the surface expression of functional receptors, depending on Ca^2+^ concentration (Zhao et al., 2009). This might contribute to the differential sensitivity of these two strains to nicotine. It is interesting to note that one of the above induced genes in LEW strain (Slc17a6 gene, coding a vesicular glutamate transporter protein) was found to be up-regulated in the VTA by extended alcohol and tobacco abuse in humans (Flatscher-Bader et al., 2008), while others (Ncam1 and Vsnl1) are among genes found to be associated with drug addiction in genome wide association studies (Li et al., 2008; Wain et al., 2015; Zhong et al., 2015).

## Conclusion

Today it is well clear that the initial goal of genetic studies to find genes with a definite contribution to substance use disorders has been elusive (Kendler et al., 2012). It is now well recognized that addiction, like other behavioral disorders, are polygenic diseases influenced by several genes affecting different neurobiological systems (Hall et al., 2013). Genes exert their influence through their action on neurotransmitter systems, drug metabolic pathways, transduction mechanisms and responses to environmental stimuli, such as stressful events, and determine individual traits, such as impulsivity, novelty/sensation seeking or stress reactivity.

From the above reviewed studies it appears that the LEW strain differs from F344 in several neurotransmitter systems either in basal conditions or following exposure to drugs of abuse. Such differences are likely at the basis of the greater sensitivity of the LEW strain to the rewarding/reinforcing properties of drugs of abuse. Moreover, LEW strain shows impulsive traits that are known to predispose to substance use disorders, but also to be a consequence of chronic drug use. Several proteins (DA receptors and transporter; endogenous opioid receptors and precursors; endocannabinoid receptors and degrading enzymes; 5-HT receptors and 5-HT transporter) found to be differentially expressed in the two strains are coded by genes whose polymorphisms have been associated with addiction to different substances in human genetic studies.

As highlighted in the introduction, gene-environment interactions are also of critical importance, contributing to the final outcome of developing a given behavioral disorder. In this regard the striking difference between LEW and F344 strains in their HPA axis activity might play an important role in coping with adverse stimuli and adapting behavior. Their different HPA axis activity might also play a significant role in response to psychostimulants, as well the greater vulnerability of LEW strain to neuroinflammatory disease might have a role in vulnerability to addiction as posited by some authors.

Thus, it would be tempting to speculate that this model might mimic several traits of a genetically vulnerable human phenotype, although keeping in mind the complexity of the human condition that could only be roughly reproduced in an animal model. Nonetheless, integration of human genetic studies by using animal models might increase our knowledge on the role of genetic contribution in substance use disorders and provide new target genes to be studied in humans helping us to find new therapeutic approaches.

## Author contributions

The author confirms being the sole contributor of this work and approved it for publication.

### Conflict of interest statement

The author declares that the research was conducted in the absence of any commercial or financial relationships that could be construed as a potential conflict of interest.
